# A small-RNA-mediated negative feedback loop controls quorum-sensing dynamics in *Vibrio harveyi*

**DOI:** 10.1111/j.1365-2958.2008.06452.x

**Published:** 2008-10-03

**Authors:** Kimberly C Tu, Christopher M Waters, Sine L Svenningsen, Bonnie L Bassler

**Affiliations:** 1 Department of Molecular BiologyPrinceton, NJ 08544-1014, USA; 2 Howard Hughes Medical Institute, Princeton UniversityPrinceton, NJ 08544-1014, USA

## Abstract

The bioluminescent marine bacterium *Vibrio harveyi* uses a cell-to-cell communication process called quorum sensing (QS) to co-ordinate behaviours in response to changes in population density. QS is accomplished through the secretion and detection of extracellular signalling molecules called autoinducers. At the centre of the *V. harveyi* QS circuit are five small regulatory RNAs called Qrr1–5 which destabilize the mRNA of *luxR*, encoding LuxR, the master transcriptional regulator of QS target genes. Here we show that LuxR directly activates transcription of *qrr*2, *qrr*3 and *qrr*4, leading to the rapid downregulation of *luxR*. The LuxR-binding sites in the promoters of *qrr*2, *qrr*3 and *qrr*4 were identified and mutated to determine the consequences of this regulatory loop on QS dynamics. Disruption of the loop delays the transition from high to low cell density, and more significantly, decreases the cell density at which the population reaches a quorum. Our results suggest that feedback is essential for optimizing the dynamics of the transitions between individual and group behaviours.

## Introduction

Bacteria respond to fluctuations in their environment by linking external sensory information to appropriate behavioural changes. The regulatory networks that tune bacteria to their environment typically transduce information to transcription factors, which control specific sets of genes in response to particular stimuli. Regulatory networks are built from recurring units called network motifs that carry out specific information-processing steps (Hartwell *et al*., 1999; Alon, 2007). Often, additional features layered onto these biological networks precisely control the dynamics of a sensory relay and enhance its robustness. For example, positive feedback loops can promote rapid transitions between distinct stable states, while negative feedback loops can accelerate response times and reduce cell-to-cell variation (Ferrell, 2002; Rosenfeld *et al*., 2002). Integration of sensory information is essential for bacterial adaptation; thus, the ecological niche of an organism likely drives the evolution of optimized network architectures.

One changing environmental parameter monitored by bacteria is cell-population density, and this is achieved through quorum sensing (QS). QS is a mechanism of chemical communication that enables bacteria to track population density by secreting and detecting extracellular signalling molecules called autoinducers (AIs) (Waters and Bassler, 2005). QS bacteria monitor the concentration of AIs as a proxy for cell number. In response to the accumulation of AIs, bacterial populations co-ordinately alter the expression of large sets of genes to carry out tasks that are presumably productive only when groups of cells act in concert.

**Fig. 1:**
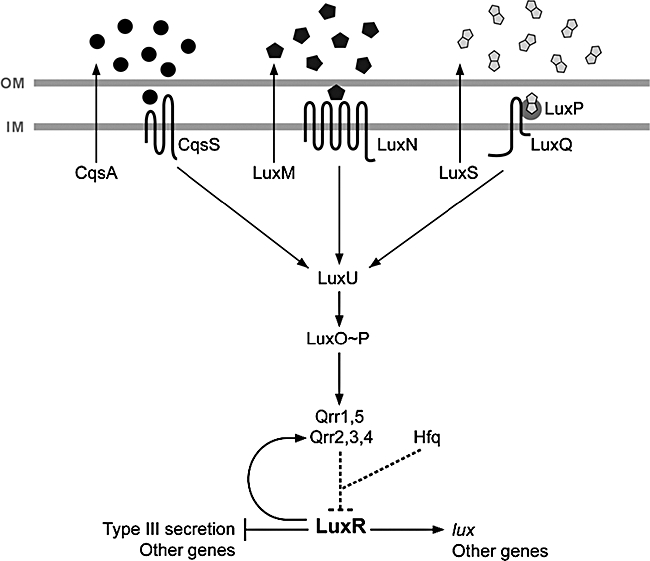
Model of the *V. harveyi* Quorum-Sensing Circuit. *V. harveyi* produces and detects three AIs and through modulation of the levels of the master transcriptional regulator, LuxR, controls downstream QS-target genes. The three AIs are: CAI-1 (circles) which binds to CqsS, HAI-1 (pentagons) which binds to LuxN and AI-2 (double pentagons) which binds to LuxPQ. At LCD, when LuxO is phosphorylated (LuxO˜P), it activates transcription of the genes encoding the five Qrr sRNAs which work in conjuction with Hfq to destabilize the mRNA of *luxR*. At HCD, when LuxO is not phosphorylated, *qrr* transcription ceases, *luxR* mRNA is stabilized and LuxR protein is produced. In a feedback loop, LuxR activates expression of *qrr*2, *qrr*3 and *qrr*4, which affects the timing of the QS transitions. OM, outer membrane; IM, inner membrane.

In the bioluminescent marine bacterium *Vibrio harveyi*, QS controls processes such as bioluminescence, type III secretion, cyclic-di-GMP production and metabolism (Fuqua *et al*., 1994; Miller and Bassler, 2001; Henke and Bassler, 2004; Waters and Bassler, 2006; Waters *et al*., 2008) (see Fig. 1 for details). *V. harveyi* synthesizes three AIs. HAI-1, *N*-(β-hydroxybutyryl) homoserine lactone, which is the strongest of the three signals, is a species-specific AI produced by the LuxM synthase (Cao and Meighen, 1989; Bassler *et al*., 1993). CAI-1 (*S*)-3-hydroxytridecan-4-one is a genus-specific signal that is produced by the CqsA synthase (Miller *et al*., 2002; Higgins *et al*., 2007). The third AI, AI-2 (2*S*,4*S*)-2-methyl-2,3,3,4-tetrahydroxytetrahydrofuran borate is an interspecies signal produced by the LuxS synthase (Bassler *et al*., 1997; Schauder *et al*., 2001; Chen *et al*., 2002). Each of the three AIs is detected by a cognate membrane-bound two-component histidine-kinase sensor: HAI-1 binds to LuxN (Bassler *et al*., 1993; Freeman *et al*., 2000), CAI-1 binds to CqsS (Miller *et al*., 2002), and AI-2 is recognized by LuxQ in conjunction with the periplasmic protein LuxP (Bassler *et al*., 1994; Neiditch *et al*., 2005; 2006).

At negligible concentrations of AIs, i.e. at low cell density (LCD), the three sensors act as kinases that transfer phosphate through LuxU to LuxO (Freeman and Bassler, 1999a,b; Lilley and Bassler, 2000). LuxO˜P activates the expression of genes encoding five highly conserved small regulatory RNAs (sRNAs) called Qrr1–5 (Tu and Bassler, 2007). The Qrrs pair with the 5′ UTR of the *luxR* mRNA and destabilize it, a process that requires the RNA chaperone Hfq (Lenz *et al*., 2004). LuxR is the master transcriptional regulator of QS genes in *V. harveyi* (Showalter *et al*., 1990; Swartzman *et al*., 1992). Thus, at LCD, when little LuxR is present, there is no QS and *V. harveyi* cells act as individuals. At high cell density (HCD), AIs accumulate and bind to their cognate sensors. This event causes the sensors to act as phosphatases, leading to dephosphorylation of LuxO. Unphosphorylated LuxO is inactive. Transcription of the sRNA-encoding genes is terminated, causing *luxR* mRNA to accumulate (Freeman and Bassler, 1999b; Lilley and Bassler, 2000). Newly produced LuxR protein activates and represses numerous genes. Most notably, LuxR activates the *luxCDABE* operon, encoding luciferase, which is required for bioluminescence (Miyamoto *et al*., 1994). Thus, at HCD, QS is initiated and *V. harveyi* cells act as a group.

Most of the regulatory components of the QS circuit have been defined in *V. harveyi*, allowing us to begin to analyse the features of the QS-signalling network that optimize *V. harveyi*'s ability to respond to differing community conditions. Here, we report the discovery of a negative feedback loop in the *V. harveyi* QS regulatory cascade involving LuxR and the Qrr sRNAs. We show that LuxR directly binds to and activates transcription of the promoters preceding *qrr*2, *qrr*3 and *qrr*4, but not *qrr*1 or *qrr*5. This leads to increased destabilization of *luxR* mRNA and downregulation of LuxR production. Mutation of the consensus LuxR-binding sites in the *V. harveyi qrr*2, *qrr*3 and *qrr*4 promoters disrupts the negative feedback loop and affects the timing of the transition from HCD to LCD mode and vice versa. In the closely related species *Vibrio cholerae*, we previously characterized a negative feedback loop consisting of HapR (the LuxR homologue) and the *V. cholerae* Qrr sRNAs (Svenningsen *et al*., 2008). However, in *V. cholerae*, the mechanism by which HapR feeds back to activate *qrr* expression is distinct from *V. harveyi*. Together, our studies suggest that LuxR/HapR-sRNA-mediated negative feedback is essential for optimizing the dynamics of the transitions between individual and group behaviours in *Vibrios*.

## Results

### LuxR binds to the promoters of *qrr2*, *qrr3* and *qrr4*

HapR, the *V. cholerae* homologue of *V. harveyi* LuxR, activates the expression of the *V. cholerae qrr* genes through an indirect mechanism (i.e. HapR does not bind the *qrr* promoters directly). The HapR-sRNA-mediated feedback loop only operates during the HCD to LCD transition, when both HapR and LuxO˜P are present, and in so doing, it functions to accelerate the transition of *V. cholerae* out of social mode into individual cell mode (Svenningsen *et al*., 2008). Because we do not know the identity of the component linking HapR to the *qrr* genes, we have only been able to examine QS behaviours in *V. cholerae* strains that are HapR^+^ or HapR^−^, but not in strains that are HapR^+^ with the negative feedback loop eliminated. *V. cholerae* and *V. harveyi* are closely related and have similar but not identical QS circuits, therefore we wondered whether *V. harveyi* has an analogous feedback loop in which LuxR activates the *V. harveyi qrr* genes. If so, we speculated that we could exploit differences between the *V. harveyi* and *V. cholerae* QS circuits to gain a further understanding of the role of this feedback loop.

**Fig. 2:**
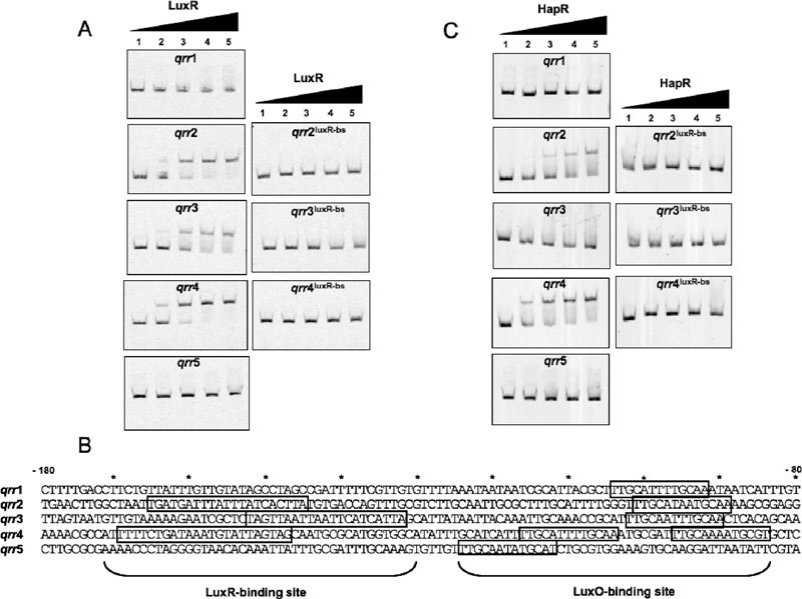
LuxR and HapR bind to the promoters of *V. harveyi qrr*2, *qrr*3 and *qrr*4 A. Gel mobility shift analyses of LuxR binding to the *V. harveyi qrr* WT promoters and the mutated *qrr*2, *qrr*3 and *qrr*4 promoters (denoted *qrr*^luxR-bs^) lacking the LuxR binding site. 10 nm of probe was used with the following concentrations of purified LuxR protein (nM) (left to right): 0, 6.25, 31.25, 62.5, 125. B. Promoter regions are shown for the five *V. harveyi qrr* genes. The consensus LuxR-binding sites (leftmost black boxes) in *qrr*2, *qrr*3 and *qrr*4 are depicted in relation to the LuxO-binding sites (rightmost black boxes). C. Gel mobility shifts for HapR binding to the *V. harveyi qrr* WT promoters and the mutated *V. harveyi qrr*2, *qrr*3 and *qrr*4 promoters (denoted *qrr*^luxR-bs^) lacking the LuxR binding site. 10 nm of probe was used with the following concentrations of purified HapR protein (nM) (left to right): 0, 25, 125, 250 500.

To test for differences between the *V. harveyi* and *V. cholerae* feedback loops, we performed gel mobility shift assays with purified LuxR protein and the upstream promoter regions of *qrr*1–5 to ascertain whether LuxR could bind to them. LuxR directly binds to the promoters of *qrr*2, *qrr*3 and *qrr*4 but not to those of *qrr*1 or *qrr*5 (Fig. 2A). Examination of the binding patterns suggests that *in vitro*, LuxR has the highest affinity for the *qrr*4 promoter, followed by *qrr*2 and then *qrr*3. In agreement with these results, the consensus LuxR-binding site, TATTGATAAATTTATCAATAA (Pompeani *et al*., 2008), is present in the promoters of *qrr*2, *qrr*3 and *qrr*4 but absent from the *qrr*1 and *qrr*5 promoters (Fig. 2B). We mutated the consensus binding site in *qrr*2, *qrr*3 and *qrr*4 by randomizing the region while maintaining the original A/T and G/C content which we term *qrr*2^luxR-bs^, *qrr*3^luxR-bs^ and *qrr*4^luxR-bs^, and this eliminated LuxR binding (Fig. 2A).

LuxR and HapR belong to the TetR family of transcriptional regulators, which share similar DNA-binding recognition sites. Importantly, the above consensus binding site is absent from all of the *V. cholerae qrr* promoters, consistent with our earlier finding that in *V. cholerae*, HapR regulation of *qrr* transcription is indirect. We wondered if HapR, while incapable of binding to the *V. cholerae qrr* promoters, could bind to the *V. harveyi qrr*2, *qrr*3 and *qrr*4 promoters as LuxR and HapR share 88% sequence identity in the DNA-binding domain and 70% sequence identity in the overall protein sequence. Gel shift analyses (Fig. 2C) show that like *V. harveyi* LuxR, HapR binds the *qrr*2, *qrr*3 and *qrr*4 promoters with the highest affinity for *qrr*4, followed by *qrr*2, and HapR shows weak affinity for *qrr*3 (Fig. 2C). Higher concentrations of HapR protein than LuxR protein were required to observe binding. However, HapR did not shift the promoters of *qrr*2, *qrr*3 and *qrr*4 when the LuxR-consensus binding site was mutated showing that HapR protein is binding specifically to the LuxR-binding site.

### LuxR is sufficient to activate transcription of *qrr2*, *qrr3* and *qrr4* in *Escherichia coli*

**Fig. 3:**
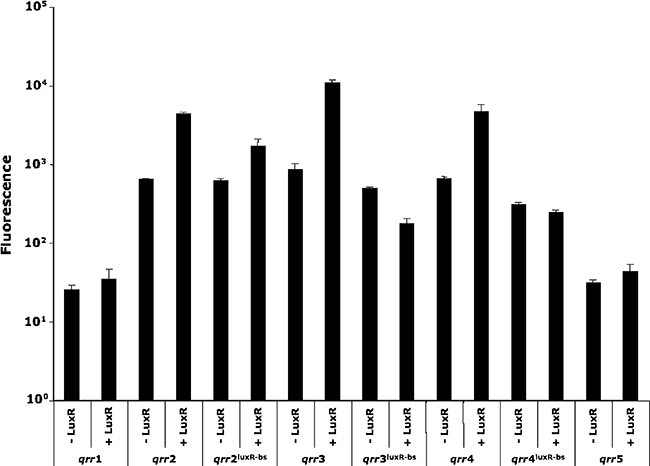
LuxR directly activates transcription of *qrr*2, *qrr*3 and *qrr*4 in *E. coli*. The constitutively active *luxO* allele, *luxO*D47E, was recombined onto the chromosome of *E. coli* strain MC4100 at the λ_att_ site. Flow cytometry was used to measure fluorescence production (in arbitrary units) from WT *V. harveyi qrr* promoter-*gfp* fusions and the *qrr* promoters with the LuxR-binding sites mutated (*qrr*^luxR-bs^). The measurements were made in the presence and absence of LuxR. *E. coli* strains: KT1646 (*qrr*1-*gfp*), KT1648 (*qrr*1-*gfp* + *luxR*), KT1529 (*qrr*2-*gfp*), KT1530 (*qrr*2-*gfp* + *luxR*), KT1535 (*qrr*2^luxR-bs^-*gfp*), KT1536 (*qrr*2^luxR-bs^-*gfp* + *luxR*), KT1531 (*qrr*3-*gfp*), KT1532 (*qrr*3-*gfp* + *luxR*), KT1614 (*qrr*3^luxR-bs^-*gfp*), KT1615 (*qrr*3^luxR-bs^-*gfp* + *luxR*), KT1533 (*qrr*4-*gfp*), KT1534 (*qrr*4-*gfp* + *luxR*), KT1539 (*qrr*4^luxR-bs^-*gfp*), KT1540 (*qrr*4^luxR-bs^-*gfp* + *luxR*), KT1647 (*qrr*5-*gfp*), KT1649 (*qrr*5-*gfp* + *luxR*). Background fluorescence was measured in the absence of LuxO˜P which averaged around 20 units (data not shown). Cultures were grown in triplicate and error bars denote standard deviation of the mean.

LuxO˜P is absolutely required for *qrr* gene expression (Lenz *et al*., 2004). Given the results in Fig. 2, we wondered whether LuxR and LuxO˜P alone are responsible for transcriptional control of the *qrr* genes or whether other factors are involved in their regulation. To investigate this, we introduced a constitutively active mutant allele of *luxO* that mimics LuxO˜P onto the chromosome of *E. coli* at the λ_att_ site. We also introduced two plasmids into this strain: one plasmid contains *luxR* under its native promoter and the second plasmid contains a *gfp*-transcriptional fusion to each of the *V. harveyi qrr* promoters. For each *qrr-gfp* fusion, we introduced constructs carrying either the wild-type (WT) LuxR-consensus-binding site or the mutated site. Figure 3 shows that in the presence of LuxO˜P transcription of *qrr*2, *qrr*3 and *qrr*4 is activated by LuxR in *E. coli* whereas *qrr*1 and *qrr*5 show no enhancement in expression in the presence of LuxR. Again, these results are supported by the bioinformatics and gel mobility shift assays in Fig. 2. In the case of the *qrr-gfp* fusions carrying mutated LuxR-binding sites, LuxR-dependent activation of expression is decreased for *qrr*2 (*qrr*2^luxR-bs^) and eliminated for *qrr*3 and *qrr*4 (*qrr*3^luxR-bs^ and *qrr*4^luxR-bs^). The gel shift assays suggest that LuxR cannot bind to the mutated *qrr*2 promoter *in vitro*; however, the experiment in Fig. 3 suggests that there apparently remains a modest LuxR effect *in vivo*. This may be due to differences in the *in vitro* and *in vivo* architecture of the *qrr*2 promoter that allows LuxR to bind *in vivo*. Together, these data indicate that in the presence of LuxO˜P, LuxR directly activates the expression of *qrr*2, *qrr*3 and *qrr*4, and the predicted LuxR-binding site is required. These results are in stark contrast to our results with *V. cholerae*, where HapR regulates *qrr* expression only through an additional unknown factor, and no activation of *qrr* expression occurs in *E. coli* carrying HapR and LuxO˜P (Svenningsen *et al*., 2008).

### Deletion of *luxR* and the LuxR-binding sites in *qrr2*, *qrr3* and *qrr4* reduce qrr expression in *V. harveyi*

**Fig. 4:**
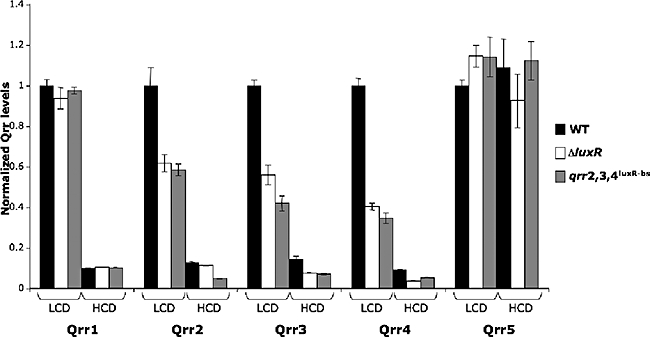
LuxR activates *qrr*2, *qrr*3 and *qrr*4 expression in *V. harveyi*. RNA was isolated from BB120 (WT; black bars), KM669 (Δ*luxR*; white bars) and KT551 (*qrr*2,3,4^luxR-bs^; grey bars) at LCD (OD_600_˜0.025) and HCD (OD_600_−1.5). Qrr levels were measured using quantitative real-time PCR. Measurements were normalized to the WT values at LCD, and fold-differences are plotted. Each sample was assayed in quadruplicate and error bars denote the standard deviation of the mean.

The above results establish a direct relationship between LuxR and *qrr*2, *qrr*3 and *qrr*4 expression. To determine whether, as in *E. coli*, LuxR activates *qrr* expression in *V. harveyi*, we measured Qrr levels at LCD and HCD using quantitative real-time PCR in WT *V. harveyi*, a Δ*luxR* strain and a strain in which the three LuxR-binding sites in the *qrr* promoters were mutated (*qrr*2,3,4^luxR-bs^). Our results are shown in Fig. 4. Each Qrr transcript is normalized to its corresponding level in the WT at LCD. First, relative to WT *V. harveyi* at LCD, Qrr levels are reduced dramatically in WT *V. harveyi* at HCD (black bars). This result is consistent with our model in which the *qrr* genes are maximally expressed at LCD when LuxO˜P is abundant, and they are minimally expressed at HCD when LuxO is unphosphorylated [Fig. 1 (Lenz *et al*., 2004; Tu and Bassler, 2007)]. Second, at LCD, Qrr2, Qrr3 and Qrr4 levels are significantly reduced in the Δ*luxR* and *qrr*2,3,4^luxR-bs^ strains compared with the WT (white bars and grey bars respectively). Third, at HCD, Qrr3 and Qrr4 levels are decreased even further in these mutant strains. These final two results confirm that LuxR feeds back to activate expression of *qrr*2, *qrr*3 and *qrr*4 *in vivo* because in the absence of LuxR or in a *V. harveyi* strain in which the LuxR-binding sites have been eliminated from the *qrr* promoters, *qrr* expression decreases. We note that Fig. 4 also confirms that *qrr*1, while controlled by cell density, is not activated by LuxR *in vivo*, and furthermore, that *qrr*5 is not subject to cell density control or LuxR-Qrr feedback regulation because Qrr5 levels are roughly identical in all strains examined. This final point is consistent with our previous studies demonstrating that QS does not control *qrr*5 expression (Tu and Bassler, 2007).

### The LuxR-sRNA feedback loop accelerates the transition from HCD to LCD

**Fig. 5:**
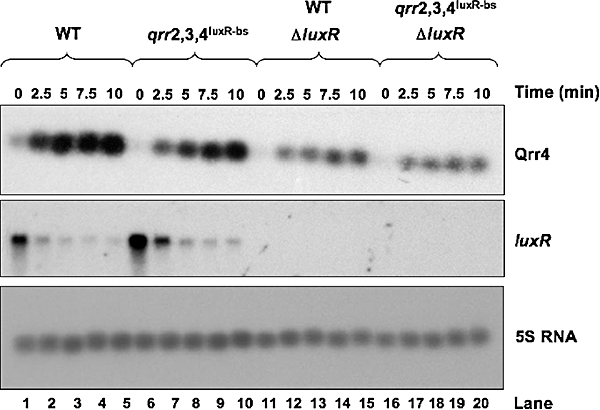
The LuxR-sRNA feedback loop affects the HCD to LCD transition. *V. harveyi* strains KT794 (Δ*luxM*, Δ*luxS*), KT797 (Δ*luxM*, Δ*luxS*, *qrr*2,3,4^luxR-bs^), KT827 (Δ*luxM*, Δ*luxS*, *luxR*::Kan) and KT829 (Δ*luxM*, Δ*luxS*, *qrr*2,3,4^luxR-bs^, *luxR*::Kan) were grown to an OD_600_−1.5 in the presence of saturating AIs (5 μM each HAI-1 and AI-2) and subsequently washed with and resuspended in fresh medium to simulate the transition from HCD to LCD. RNA was isolated at the times indicated after the wash and Qrr4 and *luxR* mRNA were analysed by Northern analysis. 5S RNA is shown as a loading control, and band intensities for *luxR* mRNA were quantified and normalized to 5S RNA. The experiment was performed twice.

To test when the *V. harveyi* feedback loop operates, we measured Qrr4 sRNA and *luxR* mRNA levels during the HCD to LCD transition in WT *V. harveyi* and compared them with those in a *V. harveyi* strain lacking the feedback loop. In this latter strain, we mutated the LuxR-binding sites in the promoters of *qrr*2, *qrr*3 and *qrr*4 (*qrr*2,3,4^luxR-bs^) to disable the feedback loop. We also compared Qrr4 levels in these two strains in a Δ*luxR* background. Additionally in all the strains, we deleted *luxM* and *luxS* encoding the AI synthases and used the addition of purified AIs to precisely control the HCD and LCD states. All the strains were grown in the presence of saturating AIs, and subsequently, the cultures were washed and resuspended in fresh medium lacking AIs to simulate an immediate transition from HCD to LCD. Figure 5 shows a Northern blot analysis of Qrr4 and *luxR* mRNA levels during the period immediately following the wash into AI-free medium. The top panel of Fig. 5 shows that, in the WT and the *qrr*2,3,4^luxR-bs^ strains, Qrr4 levels rapidly increase following the HCD to LCD transition; however, the WT strain possesses more Qrr4 than the strain lacking the LuxR-sRNA feedback loop at all time points (compare lanes 1–5 with 6–10). Quantification of the signal intensities of the Qrr4 transcript shows that immediately after the HCD to LCD transition Qrr4 accumulates twice as fast in the WT than in the strain lacking the LuxR-sRNA feedback loop. After roughly 10 min in fresh medium, Qrr4 levels in the strain lacking the feedback loop do accumulate to nearly WT levels. The middle panel shows that following removal of AIs, the *luxR* transcript is degraded more rapidly in the WT strain than in the strain lacking the feedback loop. Specifically, the *luxR* mRNA half-life is ˜2 min in the WT strain and ˜4 min in the strain lacking the feedback loop. Together, these results indicate that inactivation of the LuxR-sRNA feedback loop delays but does not abolish, the transition of *V. harveyi* from social mode to individual cell mode. Lanes 11–20 in the top panel of Fig. 5 show that Qrr4 levels are further reduced in both test strains when they lack *luxR*. This result suggests that additional factors could function through LuxR to regulate *qrr* expression in *V. harveyi*.

### The LuxR-sRNA feedback loop acts at the transition from LCD to HCD

**Fig. 6:**
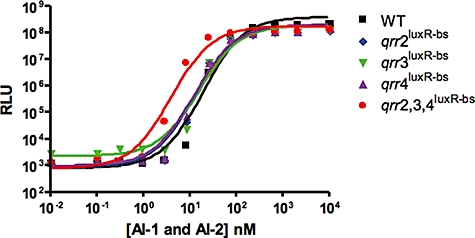
The LuxR-sRNA feedback loop affects the LCD to HCD transition. The following strains were tested for their responses to various concentrations of AIs using light production as the readout: TL27 (Δ*luxM*, Δ*luxS*, Δ*cqsS*; black squares), KT773 (TL27, *qrr*2^luxR-bs^; blue diamonds), KT770 (TL27, *qrr*3^luxR-bs^; green triangles), KT767 (TL27, *qrr*4^luxR-bs^; purple triangles), KT819 (TL27, *qrr*2,3,4^luxR-bs^; red circles). Data were fit with a variable-slope sigmoidal dose–response curve to determine the concentration of half-maximal response for each strain. RLU are counts min^−1^ ml^−1^/OD_600_.

Figure 4 shows that in the absence of the LuxR-sRNA feedback loop the levels of Qrr2, Qrr3 and Qrr4 are reduced at HCD relative to the WT. Given that the Qrr sRNAs are maximally produced at LCD, we wondered whether a reduction in Qrr levels at HCD could promote increased LuxR production, which in turn could affect the timing of HCD target gene expression. To examine this, we constructed strains that are WT for the feedback loop, that harbour a single *qrr*^luxR-bs^ mutation so that the feedback loop functions at two *qrr* genes but not the third (there are three of these mutants: *qrr*2^luxR-bs^ and *qrr*3^luxR-bs^ and *qrr*4^luxR-bs^), and the triple *qrr*2,3,4^luxR-bs^ mutant in which the feedback loop is completely eliminated. We also engineered mutations in these strains so they respond exclusively to exogenously supplied AIs by deleting *luxM* and *luxS*. We also deleted the *cqsS* gene to eliminate the response to CAI-1. Using bioluminescence as the readout, we measured the response of each strain to increasing concentrations of AIs (Fig. 6). The half-maximal effective AI concentration for WT *V. harveyi* is 19 nM. The single *qrr*^luxR-bs^ mutants respond to slightly lower AI levels (*qrr*2^luxR-bs^= 13 nM, *qrr*3^luxR-bs^= 16 nM and *qrr*4^luxR-bs^= 13 nM). The triple *qrr*2,3,4^luxR-bs^ mutant responds to significantly lower AI, 4 nM, demonstrating that the strain lacking the LuxR-sRNA feedback loop is five times more sensitive to AIs than the WT. Therefore, the LuxR-sRNA feedback does indeed control the LCD to HCD transition by preventing *V. harveyi* from prematurely entering the HCD state and misexpressing genes that are required for social behaviours. Importantly, the strains used in the experiment in Fig. 6 are all AI^−^ and thus produce no LuxR until after the addition of exogenous AI. This shows that the LuxR-sRNA negative feedback loop affects LuxR protein that is synthesized in response to AIs.

### Disruption of the LuxR-Qrr feedback loop affects quorum-sensing dynamics

One crucial aspect of the present study that distinguishes it from the earlier *V. cholerae* study is that we can excise the LuxR-sRNA-mediated feedback loop and investigate the consequences on downstream QS behaviours. To do this, we constructed *V. harveyi* strains in which only a single LuxR-binding site preceding either *qrr*2, *qrr*3 or *qrr*4 (*qrr*2^luxR-bs^, or *qrr*3^luxR-bs^ or *qrr*4^luxR-bs^) was mutated. We made a double *qrr*2,4^luxR-bs^ mutant and a triple mutant strain in which the three LuxR-binding sites were abolished (*qrr*2,3,4^luxR-bs^). This strategy allowed us to partially or completely eliminate the negative feedback loop while keeping the remainder of the QS circuit intact. We measured bioluminescence as the output of QS behaviour.

**Fig. 7:**
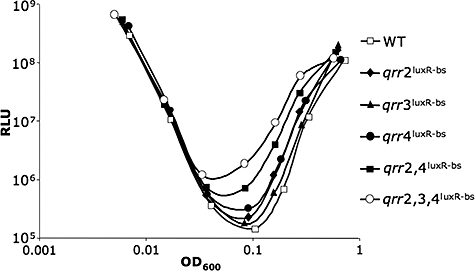
The LuxR-sRNA feedback loop affects QS dynamics Density-dependent bioluminescence was measured in the following *V. harveyi* strains: BB120 (WT, open squares), KT383 (*qrr*2^luxR-bs^; closed diamonds), KT613 (*qrr*3^luxR-bs^; closed triangles), KT311 (*qrr*4^luxR-bs^; closed circles), KT523 (*qrr*2,4^luxR-bs^; closed squares), KT551 (*qrr*2,3,4^luxR-bs^; open circles). RLU are counts min^−1^ ml^−1^/OD_600_.

In WT *V. harveyi*, expression of bioluminescence displays a hallmark QS-regulated pattern (Fig. 7, open squares): following overnight dilution of a HCD culture, light production per cell decreases dramatically. This reduction in bioluminescence is due to the dilution of the AIs below the level required to activate *lux* expression. However, as the culture grows, endogenously produced AIs increase until the critical AI threshold is reached, and light production once again commences. When only one of the three LuxR-binding sites in the *qrr* genes is mutated, *V. harveyi* exhibits modestly higher light production than WT at the transition from LCD to HCD, with the *qrr*4^luxR-bs^ mutant exhibiting the largest increase in light output (Fig. 7, closed circles), followed by the *qrr*2^luxR-bs^ mutant (Fig. 7, closed diamonds) and finally the *qrr*3^luxR-bs^ mutant (Fig. 7, closed triangles). Additionally, the transition occurs earlier in the mutants than in the WT strain, indicating that the mutant strains are responding to lower AI concentrations. This pattern parallels the pattern for the affinity of LuxR-binding to each *qrr* promoter (Fig. 2A). In the double *qrr*2,4^luxR-bs^ mutant, *V. harveyi* shows a greater increase in bioluminescence and an earlier transition than in any of the single *qrr*^luxR-bs^ mutants (Fig. 7, closed squares). Finally, in the complete absence of the LuxR-sRNA negative feedback loop (*qrr*2,3,4^luxR-bs^ triple mutant), we observe the most dramatic effect, as the strain shows an approximately 10-fold increase in bioluminescence and the earliest transition from LCD to QS mode (Fig. 7, open circles). Thus, the transition to HCD occurs with increasing rapidity in mutants that are increasingly defective in operation of the LuxR-sRNA feedback loop.

**Fig. 8:**
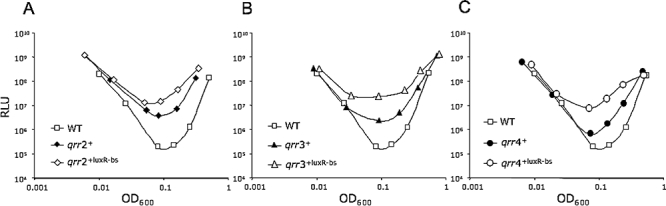
Multiple sRNAs buffer the QS circuit from perturbation. The LuxR-sRNA feedback loop was disrupted in *V. harveyi* strains containing only one *qrr* gene, and density-dependent bioluminescence was measured. WT BB120 (open squares in all panels) and the following *V. harveyi* strains were tested: A. KT280 (*qrr*2^+^, closed diamonds), KT381 (*qrr*2^+luxR-bs^, open diamonds). B. KT300 (*qrr*3^+^, closed triangles), KT548 (*qrr*3^+luxR-bs^, open triangles). C. KT281 (*qrr*4^+^, closed circles), KT353 (*qrr*4^+luxR-bs^, open circles). RLU are counts min^−1^ ml^−1^/OD_600_.

To understand the contribution of the individual LuxR-Qrr-mediated feedback loops to the QS transition, we constructed *V. harveyi* strains carrying one particular inactivating *qrr*^luxR-bs^ promoter mutation in a strain harbouring only that particular *qrr* intact. Our rationale was to examine how the feedback loop functions in the presence of multiple versus only one Qrr sRNA (Fig. 8). For comparison, we show the WT *V. harveyi* behaviour (i.e. all Qrr sRNAs are present). In the WT, maximal Qrr-mediated repression of bioluminescence expression occurs at LCD (Fig. 8, open squares in all panels). In the *qrr*2^+^ strain, in which only *qrr*2 is present, the cells produce from 10- to 100-fold more light than the WT at all stages of growth (Fig. 8A, closed diamonds), and mutating the LuxR-binding site (*qrr*2^+luxR-bs^) causes an additional ˜10-fold increase in bioluminescence (Fig. 8A, open diamonds). This same trend holds for *qrr*3 (Fig. 8B, compare closed and open triangles) and *qrr*4 (Fig. 8C, compare closed and open circles). Therefore, when there is only one Qrr sRNA functioning in the *V. harveyi* QS circuit, the LuxR-Qrr feedback loop plays a more significant role than when all the Qrr sRNAs are present, suggesting that possessing multiple Qrrs buffers the QS circuit from perturbation, and this prevents *V. harveyi* from prematurely entering the HCD, QS state.

## Discussion

Bacteria overcome the challenge of surviving in a changing environment by monitoring their environment for fluctuations in a variety of parameters, and in response to changes in these external parameters, they mount appropriate alterations in gene expression. QS allows the marine bacterium *V. harveyi* to monitor and respond to changes in the cell-population density and species-composition of the surrounding microbial community. Here, we report the existence of a negative feedback loop at the core of the *V. harveyi* QS circuit in which LuxR, the master transcriptional regulator of QS-controlled genes, directly activates the expression of the Qrr sRNAs, leading to the destabilization of the *luxR* mRNA. The LuxR-sRNA feedback loop affects the rapidity of QS-mediated changes during both QS transitions: from HCD to LCD and from LCD to HCD. Thus, the LuxR-sRNA feedback loop promotes proper timing of the gene expression programmes that underpin both individual and collective behaviours.

We have previously reported the presence of a HapR-sRNA feedback loop in the *V. cholerae* QS circuit that although indirect nonetheless accelerates the transition of the bacteria out of HCD-mode into individual cell mode (Svenningsen *et al*., 2008). Because the factor linking HapR to the regulation of the *qrr* genes remains unknown in *V. cholerae*, we were unable to uncouple the feedback loop and examine the consequences on downstream QS behaviours. Fortunately, in *V. harveyi*, as the LuxR-Qrr feedback interaction is direct, and the LuxR DNA-binding sites have been identified (Pompeani *et al*., 2008), we could disable the feedback loop and measure its effects.

At the HCD to LCD transition, the loss of the LuxR-sRNA feedback loop prevents Qrr levels from increasing to their maximum levels. Consequently, *luxR* mRNA degradation is slowed, delaying expression of genes required for individual cell behaviours (Fig. 5). Thus, in WT *V. harveyi,* the role of the LuxR-sRNA feedback loop at the HCD to LCD transition is to accelerate exit from group mode and entry into individual cell mode. At the LCD to HCD transition, disruption of the LuxR-sRNA feedback loop makes *V. harveyi* dramatically more sensitive to AIs than WT *V. harveyi*. Thus, mutants lacking the feedback loop express genes required for group behaviours at lower AI concentrations (Fig. 6). This finding implies that in the WT at the LCD to HCD transition, the role of the LuxR-sRNA feedback loop is to activate *qrr* expression, to destroy *luxR* mRNA and to control the cell density at which the population exits from individual cell mode and entry into group mode.

We note that during the LCD to HCD transition the feedback loop operates under severely limiting LuxR conditions. This means that LuxR must have high affinity for the *qrr* promoters. Accordingly, our bioinformatics analyses show that the LuxR-binding sites at the *qrr* promoters more closely resemble the LuxR-consensus binding site than do LuxR-binding sites at other known LuxR-regulated genes (Pompeani *et al*., 2008). Together, these studies suggest that when present, LuxR first binds to the *qrr* genes to initiate the feedback loop and to delay entry into QS-mode. Only later after additional LuxR protein accumulates does LuxR bind to and regulate downstream target genes.

*Vibrio harveyi* and *V. cholerae* have slightly different LuxR/HapR-sRNA feedback loops. LuxR directly activates expression of *V. harveyi qrr* genes, whereas HapR cannot activate the *V. cholerae qrr* genes. Additionally, the feedback loop in *V. harveyi* controls the transition from LCD to HCD, which does not occur in *V. cholerae*. We find that, in the *V. harveyi*Δ*luxR* strain, the delay in *qrr* expression is more drastic than is the delay in the *qrr*2,3,4^luxR-bs^ strain immediately following the HCD to LCD transition (Fig. 5). We interpret this to mean that a factor, other than LuxR, is acting on the *qrr* genes during this transition. These results suggest the possibility that the unknown component that links HapR to the *V. cholerae qrr* genes may also function in *V. harveyi*. Interestingly, the closest known relative of *V. harveyi* is the human pathogen *Vibrio parahaemolyticus*, and sequence alignment of the *V. harveyi* and *V. parahaemolyticus qrr*2, *qrr*3 and *qrr*4 promoters shows that all three LuxR-binding sites are essentially conserved. We suggest that initially the LuxR/HapR/OpaR [*V. parahaemolyticus* (McCarter, 1998)] feedback loops were indirect, and this remains the case in *V. cholerae*. Direct LuxR/OpaR feedback regulation of the *qrr* genes evolved more recently in *V. harveyi* and *V. parahaemolyticus* and likely provides some advantage for niches in which *V. harveyi* and *V. parahaemolyticus* reside, and *V. cholerae* does not.

LuxR belongs to the TetR family of transcriptional regulators, a large class of proteins particularly abundant in bacteria that are exposed to environmental changes, such as soil bacteria and plant and animal pathogens (Ramos *et al*., 2005). TetR-type proteins play important roles in adapting organisms to environmental fluctuations. These proteins are characterized by a conserved helix–turn–helix DNA-binding motif, but no sequence conservation is found in the regulatory domains, which supports their roles in responding to different stimuli (Ramos *et al*., 2005; Molina-Henares *et al*., 2006; De Silva *et al*., 2007). All of the well-characterized TetR type proteins act as repressors, where binding of an inducer molecule [such as tetracycline (Tet) to TetR] causes a conformational change, and the TetR-type protein no longer binds to DNA (Hinrichs *et al*., 1994; Orth *et al*., 1999). LuxR/HapR are the only TetR family members that appear to act as both activators and repressors (Showalter *et al*., 1990; Kovacikova and Skorupski, 2002; Pompeani *et al*., 2008). One possibility for the activation mechanism is that LuxR/HapR bends the DNA into a favourable conformation for transcription activation. Another possibility is that LuxR/HapR interacts with RNA polymerase (RNAP) holoenzyme to facilitate transcription activation. Consistent with these ideas, static and protein-induced DNA bending provides bacteria with signal integration devices for recognition of environmental inputs (Perez-Martin and de Lorenzo, 1997).

While the mechanism by which LuxR activates *qrr* expression in *V. harveyi* has yet to be determined, we know that expression of the *qrr* genes requires LuxO˜P working in conjunction with the alternative sigma factor σ^54^ (Lilley and Bassler, 2000; Lenz *et al*., 2004). σ^54^-promoters possess the unique feature that their bound activators (e.g. LuxO˜P) can function from a distance, usually 100–200 bp, through DNA looping to make contact with RNAP holoenzyme (Wigneshweraraj *et al*., 2008). The architecture of σ^54^-dependent promoters makes them ideal for allowing additional factors (e.g. LuxR) to bind and promote the optimal promoter geometry for transcriptional activation. Indeed, Fis, a small protein homodimer that generically bends DNA by 90° when bound to cognate target sites (Perez-Martin *et al*., 1994) binds to the intervening DNA sequence between the LuxO˜P and σ^54^–RNAP binding sites and is required for *qrr* activation in *V. cholerae* (and presumably in *V. harveyi*) (Lenz and Bassler, 2007). In the case of LuxR, the binding sites in the *V. harveyi qrr*2, *qrr*3 and *qrr*4 promoters are situated relatively far away, approximately 30–40 bp upstream of the LuxO˜P binding site than are typical for ancillary transcription factor binding sites, suggesting that LuxR does not function by an ordinary activation mechanism. For example, binding of LuxR to the *qrr*2, *qrr*3 and *qrr*4 promoters could block binding of a negative regulator that is conserved in *E. coli*, resulting in activation of expression (see Fig. 3). We are currently investigating the mechanism by which LuxR functions as an activator.

Small regulatory RNAs functioning in feedback loops have recently emerged as important regulatory modules in both bacteria and higher organisms (Gottesman *et al*., 2006; Tsang *et al*., 2007). MicroRNAs (miRNA) are analogous to bacterial small RNAs and confer post-transcriptional gene regulation in eukaryotic organisms by binding to complementary sequences in the 3′ UTRs of mRNAs. Large-scale computational analysis of gene expression data in mammalian cells reveals that miRNA-mediated-feedback regulation, both positive and negative, is a recurrent theme that likely enhances the robustness of gene regulation (Tsang *et al*., 2007). A double-negative feedback loop involving multiple miRNAs has recently been shown to control a neuronal cell fate decision in the nematode *Caenorhabditis elegans* (Johnston *et al*., 2005). In *Pseudomonas fluorescens* CHAO, three redundant sRNAs – RsmXYZ, sequester the RsmA and RsmE proteins, which can also feedback to activate transcription of *rsmXYZ* (Kay *et al*., 2005; 2006; Reimmann *et al*., 2005), analogous to the LuxR-Qrr feedback regulation – presented in this study. The importance of sRNA-mediated feedback loops in fast stress responses in *E. coli* and *Salmonella typhimurium* is highlighted by RybB, a sRNA that is activated by the alternative sigma factor σ^E^. RybB feeds back to downregulate σ^E^ expression, which is critical for cell-envelope homeostasis. Under membrane stress conditions, RybB is also required for rapid elimination of mRNAs encoding particular outer membrane proteins (Papenfort *et al*., 2006; Thompson *et al*., 2007). Given that positive feedback loops generally drive switch-like, irreversible processes, the specific roles of regulatory RNAs in negative feedback regulation may be one of fine-tuning, i.e. to set and maintain target protein steady states (Tsang *et al*., 2007). Compared with transcriptional repressors, regulatory RNAs may be especially effective because they control their target proteins at the post-transcriptional level and thus accelerate the response from the upstream inputs. This capacity likely leads to effective noise buffering and ensures uniform expression of the target protein within a population (Leung and Sharp, 2007).

Analyses of network topologies in *E. coli* predict that half of its transcription factors negatively auto-regulate themselves (Rosenfeld *et al*., 2002). Likewise, LuxR represses its own transcription (Chatterjee *et al*., 1996). Negative auto-regulation accelerates response times of gene expression changes and also restricts transcriptional outputs to within narrow ranges, despite widely fluctuating inputs (Rosenfeld *et al*., 2002; Seshasayee *et al*., 2006). Because LuxR is the master transcriptional regulator of a large set of QS target genes in *V. harveyi*, it is essential that LuxR levels are tightly controlled to ensure that the population elicits a precisely timed pattern of gene expression in response to changing AI concentrations. The LuxR-sRNA feedback loop described here may be especially effective in modulating gene expression changes because LuxR protein levels are negatively controlled at both the post-transcriptional and transcriptional levels. Presumably, the dual negative feedback loops that control LuxR levels ensure uniform expression of LuxR over a population of cells, which is critical for promoting synchrony in group behaviours.

## Experimental procedures

### Bacterial strains and growth conditions

All *V. harveyi* strains are derived from BB120 (Bassler *et al*., 1997) and were grown aerobically at 30°C in Luria-marine (LM) or Autoinducer Bioassay (AB) broth. *E. coli* S17–1λ*pir* was used to propagate plasmids at 37°C in LB medium. The following antibiotics were used: ampicillin, 100 μg ml^−1^; Tet, 10 μg ml^−1^; kanamycin (Kan), 100 μg ml^−1^; chloramphenicol (Cm), 10 μg ml^−1^; gentamicin, 100 μg ml^−1^ and polymyxcin B, at 50 units ml^−1^. Bacterial growth was monitored by measuring optical density at 600 nm.

### DNA manipulations

All DNA manipulations were performed using standard procedures (Sambrook *et al*., 1989). Herculase Enhanced DNA polymerase (Stratagene) was used for PCR cloning reactions, and Taq polymerase (Roche) was used for all other PCR reactions. dNTPs, restriction endonucleases and T4 DNA ligase were obtained from New England BioLabs. DNA purification kits were provided by QIAGEN. Primer sequences are available upon request. *V. harveyi* deletions were constructed using previously described methods (Datsenko and Wanner, 2000), and constructions were introduced onto the *V. harveyi* chromosome by allelic replacement (Bassler *et al*., 1993). For construction of the LuxR-bs mutants, the predicted LuxR-binding sites in the promoters of *qrr*2 and *qrr*4 were mutated to the sequence TTAGTTTGATCTGCTTAATAAA, which is not bound by LuxR as has been shown using gel shift analyses (A. Pompeani, unpubl. data). For the *qrr*3 LuxR-binding site, two bases were changed TAGTGAATTAATTCAGCATTA instead of the entire site being randomized because randomizing the entire site severely affected *qrr*3 expression. The LuxR-bs mutations for *qrr*2,3,4 were designed into the primers used in the gene replacement method as previously described (Datsenko and Wanner, 2000). *qrr-gfp* promoter fusions were cloned into pSLS3, a derivative of pCMW1 (Waters and Bassler, 2006), using the BclI and SalI restriction sites. Plasmids were transformed into *E. coli* in 0.2 cm electroporation cuvettes (USA Scientific) using a Bio-Rad Micro Pulser^TM^.

### Gel mobility shift assays

LuxR and HapR were purified with the IMPACT protein purification system (NEB) using the expression plasmid pTYB11 and the protocol described in the manufacturer's instructions. Purified LuxR was stored in 20 Tris (pH 7.5), 1 EDTA, 10 NaCL and 0.1 DTT with 20% glycerol as previously described (Waters and Bassler, 2006). DNA probes for gel mobility shift analyses were generated using 5′-tagged fluorescent primers in a standard PCR reaction that amplified 275 base pairs upstream of each *qrr* promoter. The probes were purified following agarose gel electrophoresis using the Zymoclean Gel DNA Recovery Kit (Zymo Research). Each probe (10 nM) was incubated with the indicated amount of LuxR (6.25–500 nM) and 1 μl of 1 mg ml^−1^ poly dIdC in a final volume of 20 μl at 30°C for 15 min. Gel mobility shifts were performed on a 5% TAE-polyacrylimide gel and visualized using a Storm 860 Imaging System (Molecular Dynamics).

### *gfp* expression analysis

All *gfp* expression analyses were performed on a Becton Dickinson FACSAria cell sorter, and data were analysed using FACS Diva software. For monitoring *qrr-gfp* expression in *E. coli*, cultures were grown in 2 ml LB + Tet, Cm for 12 h in triplicate at 30°C with aeration. The *E. coli* strain used in *gfp*-expression studies is MC4100. The *luxO* D47E allele was cloned into pBBR322 in the EcoRI and BamHI sites and integrated to the λ_att_ site with λInCh1 method as described previously (Boyd *et al*., 2000) to make the strain KT1190. Either *luxR* on pLAFR2 (pKM699) or the empty pLAFR2 vector was transformed into strain KT1190 along with the *qrr-gfp* reporter construct to perform the assay.

### Bioluminescence assays

*Vibrio harveyi* bioluminescence expression was measured in an assay that has been described previously (Bassler *et al*., 1993). Briefly, *V. harveyi* cultures were grown for 14 h in LM medium at 30°C with aeration. The cultures were diluted 1:5000, and light production and OD_600_ were measured every 45 min thereafter. Relative light units (RLU) are defined as counts min^−1^ ml^−1^/OD_600_. For dose–response curve experiments, cultures were grown for 14 h in AB medium and subsequently diluted 1:1000 in fresh AB medium. In a 96-well microtiter plate, 90 μl of culture was added to 5 μl of 100 μM HAI-1 and 5 μl of 100 μM AI-2, and serial dilutions were made to final concentrations of 10 pM total of both HAI-1 and AI-2. The cultures were grown for 6 h in quadruplicate, and bioluminescence and OD_600_ were measured using a Perkin Elmer EnVision plate reader.

### Quantitative real-time PCR analysis

*Vibrio harveyi* strains were grown for 14 h and subsequently diluted 1:500. Cells were pelleted at LCD (OD_600_˜0.025) and at HCD (OD_600_−1.5) and frozen at −80°C. RNA was isolated using the Ribo-Pure^TM^-Bacteria kit (Ambion/ABI). Samples were treated with DNAse I (Ambion/ABI). RNA was quantified on a NanoDrop® ND-1000 Spectrophotometer (NanoDrop Technologies). Real-time PCR analysis was carried out as described previously (Tu and Bassler, 2007). *hfq* was used as the endogenous control, and primers are available upon request.

### Northern blot analysis

*Vibrio harveyi* cultures were grown to OD_600_−1.5 in LM medium with 5 μM each HAI-1 and AI-2. The cultures were washed twice and resuspended in LM without AIs. Aliquots for RNA preparation were collected at the indicated times after the addition of fresh medium, mixed with 0.2 volume of stop solution and snap-frozen in liquid nitrogen (Papenfort *et al*., 2008). After thawing on ice, total RNA was isolated as described (Svenningsen *et al*., 2008). Northern blots were performed as described (Martin *et al*., 1989), except that single-stranded DNA probes were obtained by asymmetric PCR. Primer sequences are available upon request. Membranes were initially probed for Qrr4, then stripped by washing with 0.5% SDS for 30 min at 95°C and reprobed for LuxR mRNA, and finally stripped and reprobed for 5S RNA. Signal intensities were quantified using an Alpha Innotech FluorChem image analysis system.

## References

[b1] Alon U (2007). Network motifs: theory and experimental approaches. Nat Rev Genet.

[b2] Bassler BL, Wright M, Showalter RE, Silverman MR (1993). Intercellular signalling in *Vibrio harveyi*: sequence and function of genes regulating expression of luminescence. Mol Microbiol.

[b3] Bassler BL, Wright M, Silverman MR (1994). Multiple signalling systems controlling expression of luminescence in *Vibrio harveyi*: sequence and function of genes encoding a second sensory pathway. Mol Microbiol.

[b4] Bassler BL, Greenberg EP, Stevens AM (1997). Cross-species induction of luminescence in the quorum-sensing bacterium *Vibrio harveyi*. J Bacteriol.

[b5] Boyd D, Weiss DS, Chen JC, Beckwith J (2000). Towards single-copy gene expression systems making gene cloning physiologically relevant: lambda InCh, a simple *Escherichia coli* plasmid-chromosome shuttle system. J Bacteriol.

[b6] Cao JG, Meighen EA (1989). Purification and structural identification of an autoinducer for the luminescence system of *Vibrio harveyi*. J Biol Chem.

[b7] Chatterjee J, Miyamoto CM, Meighen EA (1996). Autoregulation of luxR: the *Vibrio harveyi* lux-operon activator functions as a repressor. Mol Microbiol.

[b8] Chen X, Schauder S, Potier N, Van Dorsselaer A, Pelczer I, Bassler BL, Hughson FM (2002). Structural identification of a bacterial quorum-sensing signal containing boron. Nature.

[b9] Datsenko KA, Wanner BL (2000). One-step inactivation of chromosomal genes in *Escherichia coli* K-12 using PCR products. Proc Natl Acad Sci USA.

[b10] De Silva RS, Kovacikova G, Lin W, Taylor RK, Skorupski K, Kull FJ (2007). Crystal structure of the *Vibrio cholerae* Quorum Sensing Regulatory Protein HapR. J Bacteriol.

[b11] Ferrell JE (2002). Self-perpetuating states in signal transduction: positive feedback, double-negative feedback and bistability. Curr Opin Cell Biol.

[b12] Freeman JA, Bassler BL (1999a). Sequence and function of LuxU: a two-component phosphorelay protein that regulates quorum sensing in *Vibrio harveyi*. Mol Microbiol.

[b13] Freeman JA, Bassler BL (1999b). A genetic analysis of the function of LuxO, a two-component response regulator involved in quorum sensing in *Vibrio harveyi*. Mol Microbiol.

[b14] Freeman JA, Lilley BN, Bassler BL (2000). A genetic analysis of the functions of LuxN: a two-component hybrid sensor kinase that regulates quorum sensing in *Vibrio harveyi*. Mol Microbiol.

[b15] Fuqua WC, Winans SC, Greenberg EP (1994). Quorum sensing in bacteria: the LuxR-LuxI family of cell density-responsive transcriptional regulators. J Bacteriol.

[b16] Gottesman S, McCullen CA, Guillier M, Vanderpool CK, Majdalani N, Benhammou J (2006). Small RNA regulators and the bacterial response to stress. Cold Spring Harb Symp Quant Biol.

[b17] Hartwell LH, Hopfield JJ, Leibler S, Murray AW (1999). From molecular to modular cell biology. Nature.

[b18] Henke JM, Bassler BL (2004). Quorum sensing regulates type III secretion in *Vibrio harveyi* and *Vibrio parahaemolyticus*. J Bacteriol.

[b19] Higgins DA, Pomianek ME, Kraml CM, Taylor RK, Semmelhack MF, Bassler BL (2007). The major *Vibrio cholerae* autoinducer and its role in virulence factor production. Nature.

[b20] Hinrichs W, Kisker C, Duvel M, Muller A, Tovar K, Hillen W, Saenger W (1994). Structure of the Tet repressor-tetracycline complex and regulation of antibiotic resistance. Science.

[b21] Johnston RJ, Chang S, Etchberger JF, Ortiz CO, Hobert O (2005). MicroRNAs acting in a double-negative feedback loop to control a neuronal cell fate decision. Proc Natl Acad Sci USA.

[b22] Kay E, Dubuis C, Haas D (2005). Three small RNAs jointly ensure secondary metabolism and biocontrol in *Pseudomonas fluorescens* CHA0. Proc Natl Acad Sci USA.

[b23] Kay E, Humair B, Denervaud V, Riedel K, Spahr S, Eberl L (2006). Two GacA-dependent small RNAs modulate the quorum-sensing response in *Pseudomonas aeruginosa*. J Bacteriol.

[b24] Kovacikova G, Skorupski K (2002). Regulation of virulence gene expression in *Vibrio cholerae* by quorum sensing: HapR functions at the aphA promoter. Mol Microbiol.

[b25] Lenz DH, Bassler BL (2007). The small nucleoid protein Fis is involved in *Vibrio cholerae* quorum sensing. Mol Microbiol.

[b26] Lenz DH, Mok KC, Lilley BN, Kulkarni RV, Wingreen NS, Bassler BL (2004). The small RNA chaperone Hfq and multiple small RNAs control quorum sensing in *Vibrio harveyi and Vibrio cholerae*. Cell.

[b27] Leung AK, Sharp PA (2007). microRNAs: a safeguard against turmoil?. Cell.

[b28] Lilley BN, Bassler BL (2000). Regulation of quorum sensing in *Vibrio harveyi* by LuxO and sigma-54. Mol Microbiol.

[b29] McCarter LL (1998). OpaR, a homologue of *Vibrio harveyi* LuxR, controls opacity of *Vibrio parahaemolyticus*. J Bacteriol.

[b30] Martin M, Showalter R, Silverman M (1989). Identification of a locus controlling expression of luminescence genes in *Vibrio harveyi*. J Bacteriol.

[b31] Miller MB, Bassler BL (2001). Quorum sensing in bacteria. Annu Rev Microbiol.

[b32] Miller MB, Skorupski K, Lenz DH, Taylor RK, Bassler BL (2002). Parallel quorum sensing systems converge to regulate virulence in *Vibrio cholerae*. Cell.

[b33] Miyamoto CM, Smith EE, Swartzman E, Cao JG, Graham AF, Meighen EA (1994). Proximal and distal sites bind LuxR independently and activate expression of the *Vibrio harveyi* lux operon. Mol Microbiol.

[b34] Molina-Henares AJ, Krell T, Eugenia Guazzaroni M, Segura A, Ramos JL (2006). Members of the IclR family of bacterial transcriptional regulators function as activators and/or repressors. FEMS Microbiol Rev.

[b35] Neiditch MB, Federle MJ, Miller ST, Bassler BL, Hughson FM (2005). Regulation of LuxPQ receptor activity by the quorum-sensing signal autoinducer-2. Mol Cell.

[b36] Neiditch MB, Federle MJ, Pompeani AJ, Kelly RC, Swem DL, Jeffrey PD (2006). Ligand-induced asymmetry in histidine sensor kinase complex regulates quorum sensing. Cell.

[b37] Orth P, Schnappinger D, Sum PE, Ellestad GA, Hillen W, Saenger W, Hinrichs W (1999). Crystal structure of the tet repressor in complex with a novel tetracycline, 9-(N,N-dimethylglycylamido)-6-demethyl-6-deoxy-tetracycline. J Mol Biol.

[b38] Papenfort K, Pfeiffer V, Mika F, Lucchini S, Hinton JC, Vogel J (2006). SigmaE-dependent small RNAs of *Salmonella* respond to membrane stress by accelerating global omp mRNA decay. Mol Microbiol.

[b39] Papenfort K, Pfeiffer V, Lucchini S, Sonawane A, Hinton JC, Vogel J (2008). Systematic deletion of *Salmonella* small RNA genes identifies CyaR, a conserved CRP-dependent riboregulator of OmpX synthesis. Mol Microbiol.

[b40] Perez-Martin J, de Lorenzo V (1997). Clues and consequences of DNA bending in transcription. Annu Rev Microbiol.

[b41] Perez-Martin J, Rojo F, de Lorenzo V (1994). Promoters responsive to DNA bending: a common theme in prokaryotic gene expression. Microbiol Rev.

[b42] Pompeani AJ, Irgon JI, Berger MF, Bulyk ML, Wingreen NS, Bassler BL (2008). The *Vibrio harveyi* master quorum-sensing regulator, LuxR, a Tet-R-type protein is both an activator and a repressor: DNA recognition and binding specificity at target promoters. Mol Microbiol.

[b43] Ramos JL, Martinez-Bueno M, Molina-Henares AJ, Teran W, Watanabe K, Zhang X (2005). The TetR family of transcriptional repressors. Microbiol Mol Biol Rev.

[b44] Reimmann C, Valverde C, Kay E, Haas D (2005). Post-transcriptional repression of GacS/GacA-controlled genes by the RNA-binding protein RsmE acting together with RsmA in the biocontrol strain *Pseudomonas fluorescens* CHA0. J Bacteriol.

[b45] Rosenfeld N, Elowitz MB, Alon U (2002). Negative autoregulation speeds the response times of transcription networks. J Mol Biol.

[b46] Sambrook J, Fritsch EF, Maniatis T (1989). Molecular Cloning: A Laboratory Manual.

[b47] Schauder S, Shokat K, Surette MG, Bassler BL (2001). The LuxS family of bacterial autoinducers: biosynthesis of a novel quorum-sensing signal molecule. Mol Microbiol.

[b48] Seshasayee AS, Bertone P, Fraser GM, Luscombe NM (2006). Transcriptional regulatory networks in bacteria: from input signals to output responses. Curr Opin Microbiol.

[b49] Showalter RE, Martin MO, Silverman MR (1990). Cloning and nucleotide sequence of *luxR*, a regulatory gene controlling bioluminescence in *Vibrio harveyi*. J Bacteriol.

[b50] Svenningsen SL, Waters CM, Bassler BL (2008). A negative feedback loop involving small RNAs accelerates *Vibrio cholerae*'s transition out of quorum-sensing mode. Genes Dev.

[b51] Swartzman E, Silverman M, Meighen EA (1992). The *luxR* gene product of *Vibrio harveyi* is a transcriptional activator of the lux promoter. J Bacteriol.

[b52] Thompson KM, Rhodius VA, Gottesman S (2007). SigmaE regulates and is regulated by a small RNA in *Escherichia coli*. J Bacteriol.

[b53] Tsang J, Zhu J, van Oudenaarden A (2007). MicroRNA-mediated feedback and feedforward loops are recurrent network motifs in mammals. Mol Cell.

[b54] Tu KC, Bassler BL (2007). Multiple small RNAs act additively to integrate sensory information and control quorum sensing in *Vibrio harveyi*. Genes Dev.

[b55] Waters CM, Bassler BL (2005). QUORUM SENSING: cell-to-cell communication in bacteria. Annu Rev Cell Dev Biol.

[b56] Waters CM, Bassler BL (2006). The *Vibrio harveyi* quorum-sensing system uses shared regulatory components to discriminate between multiple autoinducers. Genes Dev.

[b57] Waters CM, Lu W, Rabinowitz JD, Bassler BL (2008). Quorum Sensing Controls Biofilm Formation in *Vibrio cholerae* through Modulation of Cyclic Di-GMP Levels and Repression of. Vpst J Bacteriol.

[b58] Wigneshweraraj S, Bose D, Burrows PC, Joly N, Schumacher J, Rappas M (2008). Modus operandi of the bacterial RNA polymerase containing the sigma54 promoter-specificity factor. Mol Microbiol.

